# Vitamin D ameliorates asthma‐induced lung injury by regulating HIF‐1α/Notch1 signaling during autophagy

**DOI:** 10.1002/fsn3.2880

**Published:** 2022-04-19

**Authors:** Chaowen Huang, Ming Peng, Jinzhai Tong, Xueying Zhong, Jun Xian, Liandi Zhong, Jiongrui Deng, Yanming Huang

**Affiliations:** ^1^ Department of Pulmonary and Critical Care Medicine Jiangmen Institute of Respiratory Diseases Jiangmen Central Hospital, Jiangmen Hospital of Sun Yat‐sen University Jiangmen China

**Keywords:** asthma, autophagy, HIF‐1α/Notch1, LC 3B, vitamin D

## Abstract

Herein, we aimed to determine the effect of vitamin D (Vit D) and underlying mechanisms on asthma‐induced lung injury via regulation of HIF‐1α/Notch1 (hypoxia‐inducible factor 1 alpha/neurogenic locus notch homolog protein 1) signaling during autophagy. We established an asthma mouse model using respiratory syncytial virus (RSV) nasal drop combined with ovalbumin (OVA) atomization. Mice were treated with different Vit D concentrations. Pathological changes and cell apoptosis were examined using hematoxylin–eosin (HE) staining and TUNEL (terminal deoxynucleotidyl transferase‐mediated deoxyuridine triphosphate (dUTP) nick end‐labeling) assay, respectively. Additionally, periodic acid–Schiff (PAS) and Masson's trichrome staining solutions were used to examine changes in lung tissue. Immunofluorescence determined LC 3B (microtubule‐associated protein 1 light chain 3B) expression in lung tissues, whereas western blotting and immunohistochemistry were used to evaluate other proteins, including HIF‐1α and Notch1. Compared with the normal group, the asthma model group exhibited pathological lung tissue deterioration, elevated fibrosis, increased apoptosis cell numbers, and upregulated autophagy. Vitamin D supplementation ameliorated pathological changes and fibrosis in the lung tissue. Furthermore, Vit D treatment significantly suppressed apoptotic cell numbers and autophagy while enhancing the HIF‐1α/Notch1 pathway. Given the HIF‐1α/Notch1 agonistic activity, Vit D treatment inhibited apoptosis cell numbers, which were increased following asthma‐induced upregulation of autophagy. Vitamin D improved asthma‐induced lung tissue injury by suppressing autophagy via regulation of HIF‐1α/Notch1 signaling in vivo.

## Graphical abstract

Vitamin D improved asthma‐induced lung tissue injury by suppressing autophagy via regulation of HIF‐1α/Notch1 (hypoxia‐inducible factor 1 alpha/neurogenic locus notch homolog protein 1) signaling in vivo.

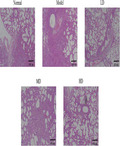

## 1 INTRODUCTION

Bronchial asthma is a respiratory disease characterized by chronic airway inflammation, airway remodeling, and airway hyperresponsiveness as primary pathological features. In recent years, childhood asthma has become one of the most common chronic diseases of the respiratory system, evolving into a global public health challenge (Feitosa et al., 2011). Respiratory syncytial virus (RSV) infection can directly impact acute onset or exacerbation of childhood asthma (Mikhail & Grayson, 2019). Currently, long‐term inhaled corticosteroids are the preferred treatment for childhood asthma. Although the illness can be greatly alleviated in children, long‐term corticosteroid therapy can cause hormone resistance or dependence, leading to related complications (Ménard et al., 2018). Accordingly, identifying effective and safe drugs for treating asthma has become a hotspot for clinical investigators.

During autophagy, lysosomes consume damaged organelles and macromolecular proteins to complete self‐metabolism and renewal of organelles (Zeki et al., 2016). Autophagy is a key regulator in the pathogenesis of asthma, affecting innate immune response and promoting programmed cell death. Furthermore, it is a critical pathway to protect the human body from virus invasion. During pathogen infection, host cells eliminate pathogens through the autophagic process (Li et al., 2020), indicating that autophagy could be a novel target for preventing and treating asthma. Dendritic cells (DCs), as the most potent antigen‐presenting cells, act at the initial stage of asthma. Following human RSV infection, autophagy may play a role in the occurrence and development of asthma by affecting pathophysiological processes such as viral replication and DC activation (Morris et al., 2011). Studies have shown that increased Notch1 (neurogenic locus notch homolog protein 1) expression can induce autophagy (Tao et al., 2018; Xu et al., 2019), whereas hypoxia‐inducible factor 1α (HIF‐1α) has a positive regulatory effect on Notch1 (Wang et al., 2021; Zhang et al., 2019).

According to a study by Ramirez et al. (2010), vitamin D (Vit D) can inhibit profibrosis and epithelial–mesenchymal transition induced by transforming growth factor‐β (TGF‐β) in epithelial cells and fibroblasts. In addition, studies have shown that Vit D levels in the body can be positively correlated with lung function (Zosky et al., 2011). Nevertheless, reports on underlying mechanisms through which Vit D improves asthma via autophagy regulation remain unclear. Consequently, in the present study, we established a mouse model of asthma using RSV combined with ovalbumin (OVA) to explore the effect of Vit D on airway responsiveness via regulation of autophagy and related mechanisms. Herein, we aimed to elucidate a new theoretical and practical basis for clinical prevention and treatment of asthma.

## 2 MATERIALS AND METHODS

### 2.1 Animals and viruses

In total, 70 specific pathogen‐free (SPF) female BALB/c mice, aged 6–8 weeks and weighing 18–20 g, were procured from Hunan SJA Laboratory Animal Co., Ltd. (animal Production Qualification Certificate No.: SCXK [Hunan] 2016‐0002). RSV long strain and human laryngeal carcinoma epithelial cells (Hep‐2) were provided by the Institute of Virology, Wuhan University School of Medicine. The experimental protocol was approved by the Ethics Committee of the Jiangmen Central Hospital (approval No.: 20200810‐02).

### 2.2 Reagents

The following reagents were used in the present study: OVA (batch No.: 9006‐59‐1; Solarbio Life Science); acetylcholine chloride (batch No.: 2260‐50‐6; Selleck); hematoxylin–eosin (HE) staining solution (batch No.: P032IH; Auragene Bioscience Co.); Masson and periodic acid–Schiff (PAS) staining solutions (batch Nos. G1285 and G1243, respectively; Solarbio Life Science); interleukin (IL)‐6, IL‐10, and IL‐17 enzyme‐linked immunoassay (ELISA) kits; (Shanghai Jingtian Biotechnology Co., Ltd.); TUNEL kit (cat. no. KGA703; KeyGen Biotech); rabbit antimouse primary antibody LC 3B (cat. no. ab225383; Abcam), Beclin1 (cat. no. ab207612; Abcam), P62 (cat. no. ab91526; Abcam), HIF‐1α (cat. no. ab51608; Abcam), and Notch1 (cat. no. ab276343; Abcam); GAPDH (cat. no. ab181602; Abcam); goat antirabbit secondary antibody (batch No. SA00001‐2; Proteintech); Trizol reagent (batch No. 15596026; Ambion, Inc.); reverse transcription kit (batch no.: K1622; Thermo Scientific); real‐time fluorescent quantitative polymerase chain reaction (real‐time qPCR) kit (batch No. PA0025; Shanghai Jinan Biotechnology Co., Ltd.).

### 2.3 Instruments

The following instrumentation was used in the present study: Ultrasonic nebulizer (model S888E; Nanjing Daofen Electronics Co., Ltd.); small animal ventilator and airway resistance and lung compliance analysis software (model nos. DHX‐50 and WESTERN BLOTP, respectively; BUXCO); desk centrifuge (model 12008007; Jintan Earth Automation Instrument Factory); fluorescence quantitative PCR instrument (model CFX; Bio‐Rad); electrophoresis apparatus and membrane transfer apparatus (Beijing Liuyi Biotechnology Co., Ltd., model nos. DYY‐6C and DYCZ‐40D, respectively).

### 2.4 Methods

#### 2.4.1 Modeling and animal grouping

In total, 70 SPF female BALB/c mice were randomly divided into normal (*n* = 20) and model (*n* = 50) groups. The model was established, as previously described (Uhl et al., 2010). Two days prior to experimentation, mice in the model group were administered a 0.05 ml RSV nasal drip and 0.25 ml of 1% OVA intraperitoneally; the normal group was administered an equal volume of Hep‐2 cells nasal drip and subcutaneous injection of normal saline. From day 9 of experimentation, mice in the model group received atomized 1% OVA for 30 min in a partially closed atomization inhalation chamber constructed in‐house; mice in the normal group received atomized normal saline at the same volume, once every other day for 2 weeks. After nebulization, mice in the model group exhibited shortness of breath, wheezing, tense abdominal muscles, and unsteadiness while standing, indicating the successful establishment of the asthma model. Then, 10 mice were randomly selected from normal and model groups to measure airway reactivity, as shown in Table 1. We observed that airway responsiveness was significantly higher in the model group mice than in the normal group, indicating that the model was successfully established. The normal group and the model mice were randomly divided into 5 groups, with 10 mice in each group, namely the normal group, model group, low‐dose group (LD), medium‐dose group (MD), and high‐dose group (HD).

**TABLE 1:** Comparison of airway hyperresponsiveness (AHR) in two groups of mice

Group	Acetylcholine (mg/ml)				
	0.00	6.25	12.50	25.00	50.00
Normal	2.03 ± 0.11	3.64 ± 0.26	5.87 ± 0.50	8.70 ± 0.54	11.52 ± 0.68
Model	2.34 ± 0.12	10.84 ± 0.68*	13.00 ± 0.78*	20.55 ± 0.72**	28.62 ± 1.13***

*p < .05, **p < .01, ***p < .001, compared with Normal group.

#### 2.4.2 Dosing

As previously reported (Morello et al., 2018), mice in the HD, MD, and LD Vit D groups were administered 1000 IU/kg/day, 500 IU/kg/day, and 100 IU/kg/day by oral gavage, respectively, and were intraperitoneally injected 0.9% normal saline (10 ml/kg). Both normal and model groups were intragastrically injected with the same volume of normal saline once daily for two consecutive weeks.

#### 2.4.3 Airway responsiveness measurement

Twenty‐four hours after the last administration, mice were placed in the body tracing box and connected to the ventilator after endotracheal intubation under anesthesia. Ventilator parameters were adjusted to a frequency of 75 times/min and tidal volume of 8 ml/kg. Changes in the initial airway pressure, flow rate, and tidal volume were recorded. Aerosol inhalation of 0.1 ml of acetylcholine at different concentrations (0, 6.25, 12.5, 25, and 50 μg/kg) was performed. Data were collected from 5 s to 1 min after acetylcholine inhalation, and airway hyperreactivity (AHR) was calculated.

#### 2.4.4 Examination of HE‐, Masson‐, and PAS‐stained tissues

After the experiment, the right middle lung of mice was immersed in paraformaldehyde and fixed for 24 h; the remaining lung tissues were placed in liquid nitrogen for later use. Conventional paraffin embedding and sectioning were performed, followed by HE, Masson, and PAS staining, to examine histopathological changes, airway collagen deposition, and mucus reserves. The Image‐Pro Plus 6.0 system was utilized to quantitatively analyze Masson and PAS staining results.

#### 2.4.5 TUNEL detection

This experiment was performed according to the TUNEL (terminal deoxynucleotidyl transferase‐mediated deoxyuridine triphosphate (dUTP) nick end‐labeling) kit instructions. Briefly, paraffin sections were dewaxed using xylene I and xylene II, dehydrated with gradient alcohol, and then thrice rinsed with phosphate‐buffered saline (PBS) for 5 min. Next, microwave repair was performed with citric acid buffer (pH 6.0) for 15 min, cooled at room temperature, digested with protease K at 37°C for 30 min, and thrice rinsed with PBS for 5 min. Subsequently, the specimens were sealed with 3% hydrogen peroxide (H_2_O_2_) for 10 min and thrice rinsed with PBS for 5 min. Deoxyribonuclease I (DNase I) reaction solution was used to prepare positive control films, which were reacted for 10 min and thrice rinsed with PBS for 5 min. Terminal deoxynucleotidyl transferase (TdT) enzyme reaction solution (50 μl) was added to the experimental group and positive control films, reacted at 37°C for 60 min, and thrice rinsed with PBS for 5 min. Next, streptavidin–horseradish peroxidase (HRP) working liquid was added, the reaction was maintained at 37°C for 30 min in darkness and then thrice rinsed with PBS for 5 min. Diaminobenzidine (DAB) working liquid was prepared, and an appropriate amount was added for color development until positive control showed good color development; then, the color reaction was terminated. Finally, nuclei were restained and sealed with neutral gum.

#### 2.4.6 Immunohistochemical staining

Briefly, paraffin sections were dewaxed using xylene I and xylene II, dehydrated with gradient alcohol, and thrice rinsed with PBS buffer for 3 min. Subsequently, microwave repair was performed with citric acid buffer (pH 6.0) for 15 min, cooled at room temperature, and thrice rinsed with PBS buffer for 5 min. Next, an appropriate amount of endogenous peroxidase blocker was added, incubated at room temperature for 10 min, and thrice washed with PBS buffer for 3 min. P62 (1:200) and Beclin1 antibodies (1:200) were then added and incubated in a wet box for 2 h and thrice washed with PBS buffer for 3 min. An appropriate amount of reaction enhancement solution was added, incubated at room temperature for 20 min, and thrice washed with PBS buffer for 3 min. Subsequently, an appropriate amount of enhanced HRP‐conjugated goat antirabbit immunoglobulin G (IgG) polymer was added, incubated at room temperature for 20 min, and thrice washed with PBS buffer for 3 min. The color reaction was terminated after 30 s of DAB color development. Nuclei were restained with hematoxylin and sealed with dehydrated, transparent neutral gum.

#### 2.4.7 Immunofluorescence determination of LC 3B expression

After dewaxing, dehydration, and high‐ and low‐temperature antigen retrieval, goat serum blocking antibody was added to the specimen, and the primary antibody LC 3B was added dropwise and diluted with PBS (1:100), until the tissue was completely covered. The specimens were maintained in the wet box at 4°C overnight. Cy3‐labeled goat antirabbit fluorescent secondary antibody was added until the tissue was completely covered, diluted with PBS (1:200) while avoiding light, and incubated at room temperature for 60 min. 4′,6‐diamidino‐2‐phenylindole (DAPI) was added until the tissue was completely covered to restain the nucleus. Next, half a drop of antifluorescence quench agent was added using a glue head dropper, and a cover glass was placed to seal the film. Finally, sections were examined under a microscope and photographed for documentation.

#### 2.4.8 ELISA detection

Blank control, negative control, and positive control wells were established to add samples, followed by incubation at 37°C for 60 min before washing. Next, 0.1 ml of freshly diluted HRP‐conjugated antibody was added to each reaction well, incubated at 37°C for 30–60 min, and then washed. Subsequently, 0.1 ml of the temporarily prepared 3,3′,5,5′‐tetramethylbenzidine (TMB) substrate solution was added for color development and mixed; color development was performed at 37°C for 10 min in the dark. Next, 0.05 ml of 2 mol/L sulfuric acid was added to terminate the reaction. After blank wells were corrected to zero, the absorbance of each well was measured at a wavelength of 450 nm using a microplate colorimeter. The determination should be performed within 10 min of adding the stopping solution. Finally, the linear regression equation of the standard curve and corresponding sample concentration were calculated.

#### 2.4.9 Real‐time PCR

Total RNA from lung tissue was extracted by employing the Trizol method, using 1 μl to determine sample concentration, with 1 μg RNA reverse‐transcribed into complementary DNA (cDNA) using a 20 μl system. Based on this template, a two‐step real‐time PCR quantitative method was established. Predenaturation was performed at 95°C for 10 min, followed by each step of denaturation at 95°C for 15 s, and annealing and extension at 60°C for 60 s. A total of 40 cycles were performed, and the fluorescence value was read during each extension phase. The results were measured using dissolution and amplification curves, and the 2^−ΔΔCt^ method was employed to analyze results with glyceraldehyde‐3‐phosphate dehydrogenase (GAPDH) as the internal reference. Specific primers were synthesized by Changsha Dimensional World Biological Science and Technology Co., Ltd., as shown in Table 2.

**TABLE 2:** The primer sequences

Gene name	F:(5′‐3′)	R:(5′‐3′)
HIF‐1α	GGGAGAAAATCAAGTCGTGC	AGCAAGGAGGGCCTCTGATG
Notch1	AGCAAGGAGGCCAAGGACC	GACCCGCCCACAGTGAAA
LC 3B	GGCAACAGCAACAGGAA	GGGGATGGTCTGAGTGTC
P62	ACAGGAAGGCCCCACAG	AGAGCAGCCCCGATGTC
Beclin1	TAGCCGACCGGGAAGTA	CGACGCTCTTCACCTCA
GAPDH	CGGCAAGTTCAACGGCACAGT	CGCTCCTGGAAGATGGTGATGG

#### 2.4.10 Western Blot method (WB)

Total protein was extracted from lung tissue, and protein quantification was performed by the bicinchoninic acid method. Briefly, 50 μg of total protein from each group was added to sodium dodecyl sulfate loading buffer at a ratio of 1:4. After heating for 5 min, the samples were subjected to electrophoresis, followed by membrane transfer and sealing for 1 h. HIF‐1α (1:200) and Notch1 (1:200) rabbit antipolyclonal antibodies and internal reference GAPDH (1:5000) were incubated overnight and washed with Tris‐buffered saline containing 0.01% Tween‐20 (TBST) 3 times for 10 min each. Subsequently, the secondary antibody (1:6000) was added, incubated for 1 h, and washed with TBST 3 times for 10 min each. After scanning, Quantity One gray analysis software was used, and the corresponding target protein expression index was determined as the ratio of the target protein band to the internal reference GAPDH.

### 2.5 Statistical analysis

Data analysis was performed using SPSS 20.0 (IBM Corp.). Data values are expressed as mean ± standard deviation (SD), and a normal distribution test was performed. If the data satisfied normal distribution, one‐way analysis of variance (ANOVA) was used to compare the means between any two groups, and the least significant difference (LSD) test was used to examine the homogeneity of variance. A value of *p* < .05 was used to establish a statistically significant difference.

## 3 RESULTS

### 3.1 Effect of Vit D on the pathological morphology of lung tissue in asthmatic mice

**FIGURE 1:**
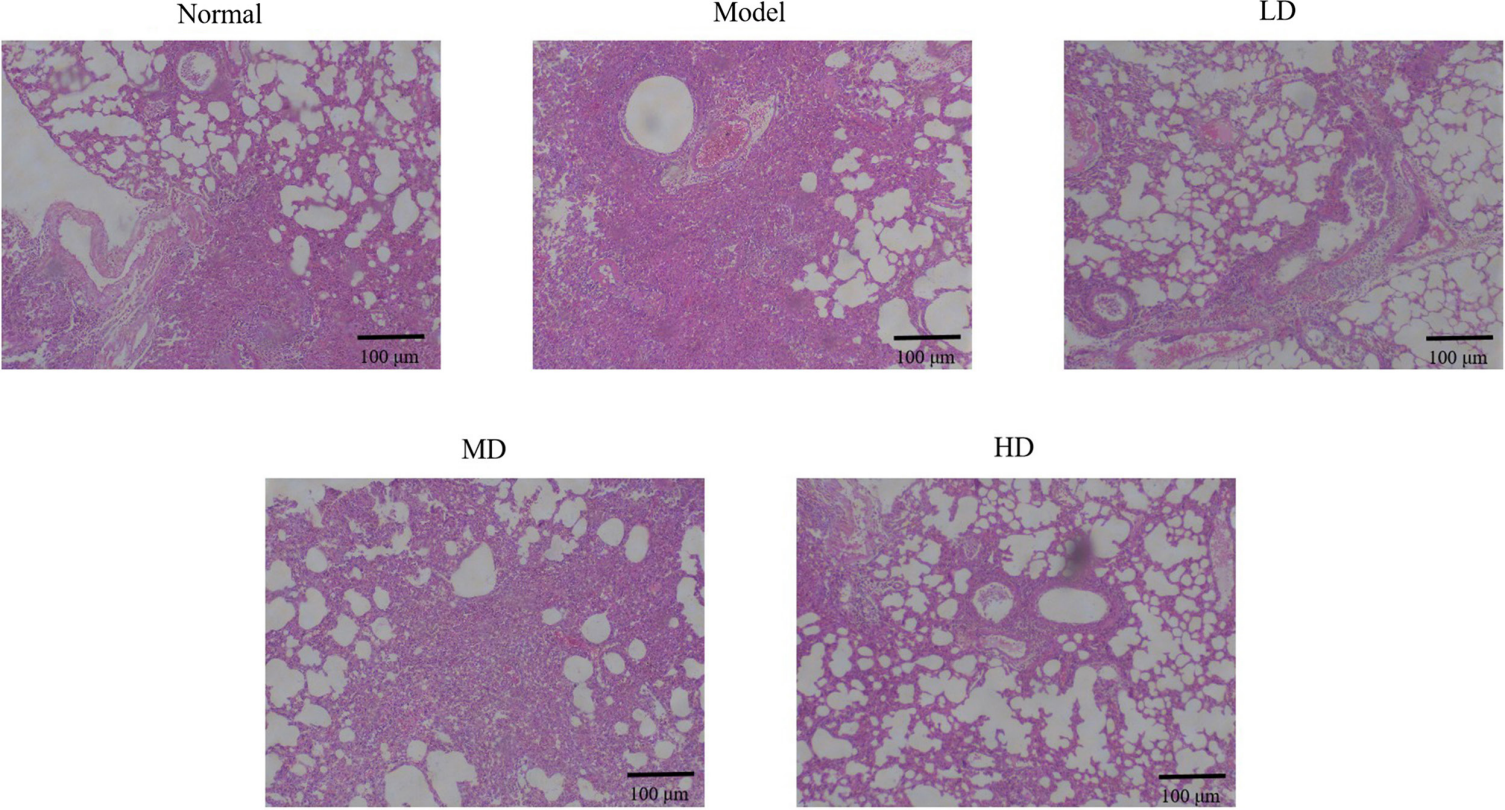
Effect of vitamin D (Vit D) on lung tissue morphology of asthmatic mice (hematoxylin–eosin [HE], ×100). Normal: Mice treated with normal saline; Model: asthmatic model mice treated with normal saline; LD: asthmatic model mice treated with low‐dose Vit D (100 IU/kg/day); MD: asthmatic model mice treated with middle‐dose Vit D (500 IU/kg/day); HD: asthmatic model mice treated with high‐dose Vit D (1000 IU/kg/day)

In the normal group, mice displayed regular bronchial and vascular morphological features without obvious inflammatory cell infiltration. In contrast, mice in the model group exhibited narrow bronchial lumen, swelling or shedding of epithelial cells, marked inflammatory infiltration around blood vessels and bronchi, notable epithelial goblet cell proliferation, and thickened airways. Compared with the model group, LD, MD, and HD Vit D groups showed improved lung tissue damage and inflammatory cell infiltration, similar to the normal group. Moreover, the HD Vit D group exhibited the best effect, as shown in Figure 1.

### 3.2 Effect of Vit D on serum levels of IL‐6, IL‐10, and IL‐17 in the asthmatic mice

Compared with the normal group, serum levels of IL‐6 and IL‐17 were significantly increased in the model group (*p* < .001), while that of IL‐10 was significantly decreased (*p* < .001). Compared with the model group, IL‐6 and IL‐17 were significantly reduced in the LD, MD, and HD Vit D groups (*p* < .05), whereas IL‐10 levels were significantly elevated (*p* < .05), as shown in Table 3.

**TABLE 3:** Effects of Vit D on the contents of interleukin 6 (IL‐6), interleukin 10 (IL‐10), and interleukin 17 (IL‐17) in serum of mice (mean ± SD)

Group	Dose (IU/kg)	IL‐3 (pg/ml)	IL‐10 (pg/ml)	IL‐17 (pg/ml)
Normal	–	14.61 ± 0.43	58.73 ± 1.87	23.18 ± 1.36
Model	–	35.59 ± 0.34***	31.53 ± 1.19***	63.50 ± 2.26***
LD	100	32.56 ± 0.32^#^	35.23 ± 0.84^#^	58.73 ± 2.89^#^
MD	500	26.60 ± 0.50^##,$^	44.64 ± 1.69^##,$^	45.26 ± 2.23^##,$^
HD	1000	18.04 ± 0.48^###,$$,&^	55.49 ± 1.07^###,$$,&^	29.16 ± 2.47^###,$$,&^

***p < .001, compared with Normal; ^#^ *p* < .05, ^##^ *p* < .01, ^###^ *p* < .001, compared with Model; ^$^ *p* < .05, ^$$^ *p* < .01, compared with LD group; ^&^ *p* < .05, compared with MD group.

### 3.3 Effect of Vit D on airway mucus reserve in the asthma mouse model

**FIGURE 2:**
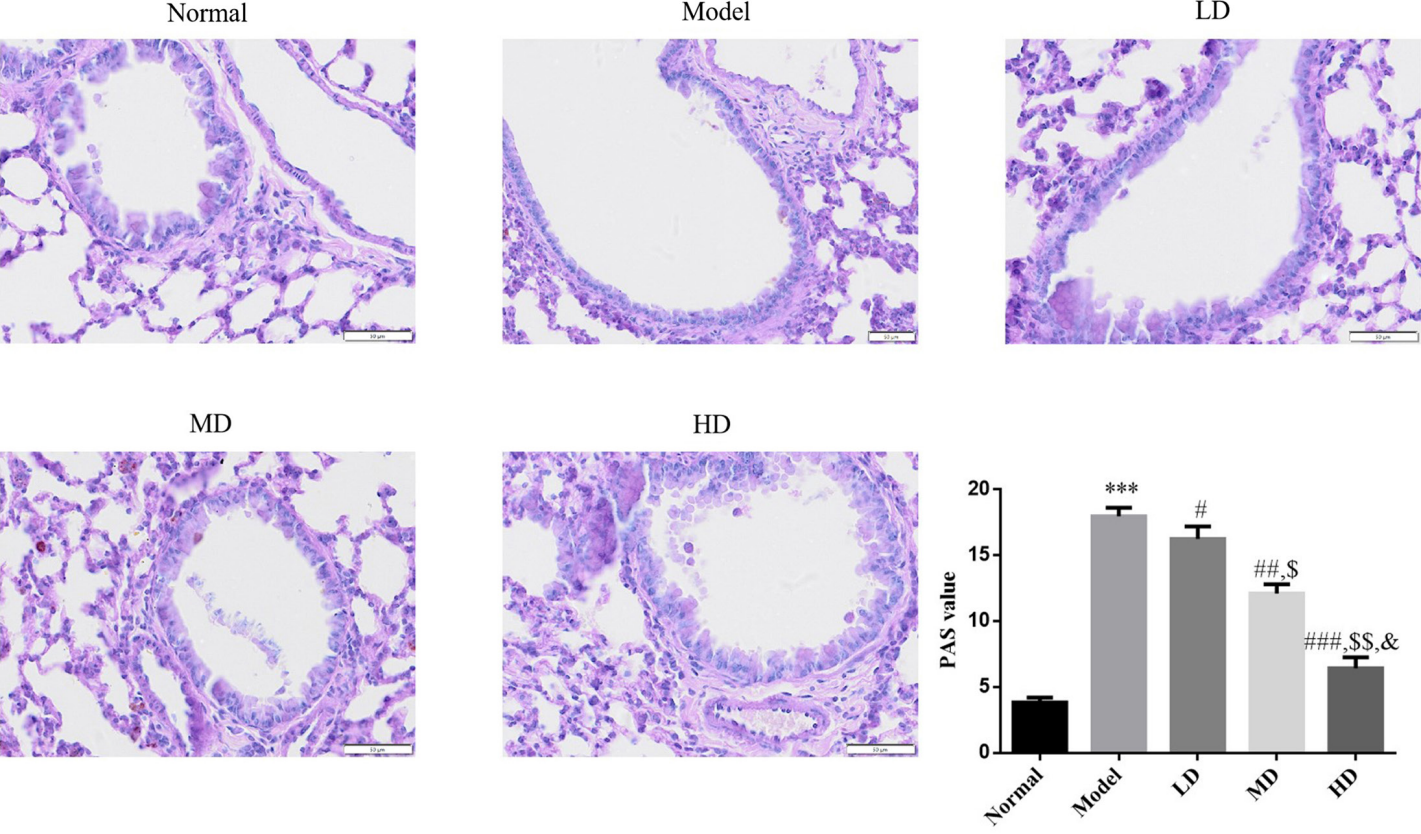
Effect of vitamin D (Vit D) on airway mucus reserve in mice (periodic acid–Schiff [PAS], ×200). Normal: Mice treated with normal saline; Model: asthmatic model mice treated with normal saline; LD: asthmatic model mice treated with low‐dose Vit D (100 IU/kg/day); MD: asthmatic model mice treated with middle‐dose Vit D (500 IU/kg/day); HD: asthmatic model mice treated with high‐dose Vit D (1000 IU/kg/day). ***p < .001, compared with Normal; ^#^ *p* < .05, ^##^ *p* < .01, ^###^ *p* < .001, compared with Model; ^$^ *p* < .05, ^$$^ *p* < .01, compared with LD group; ^&^ *p* < .05, compared with MD group

Compared with the normal control group, red‐stained positive cells were significantly increased in the lung tissue of model control group mice, whereas PAS‐stained positive cells were decreased in the LD, MD, and HD Vit D groups. Quantitative analysis of the PAS red‐stained positive area showed that the airway mucus reserve index was significantly elevated in the model control group (*p* < .001) compared with the normal control group; Vit D‐treated groups significantly reduced the mucus reserve index of asthmatic mice (*p* < .05). The HD Vit D group exhibited a superior effect, as shown in Figure 2.

### 3.4 Effect of Vit D on airway collagen deposition in asthmatic mice model

**FIGURE 3:**
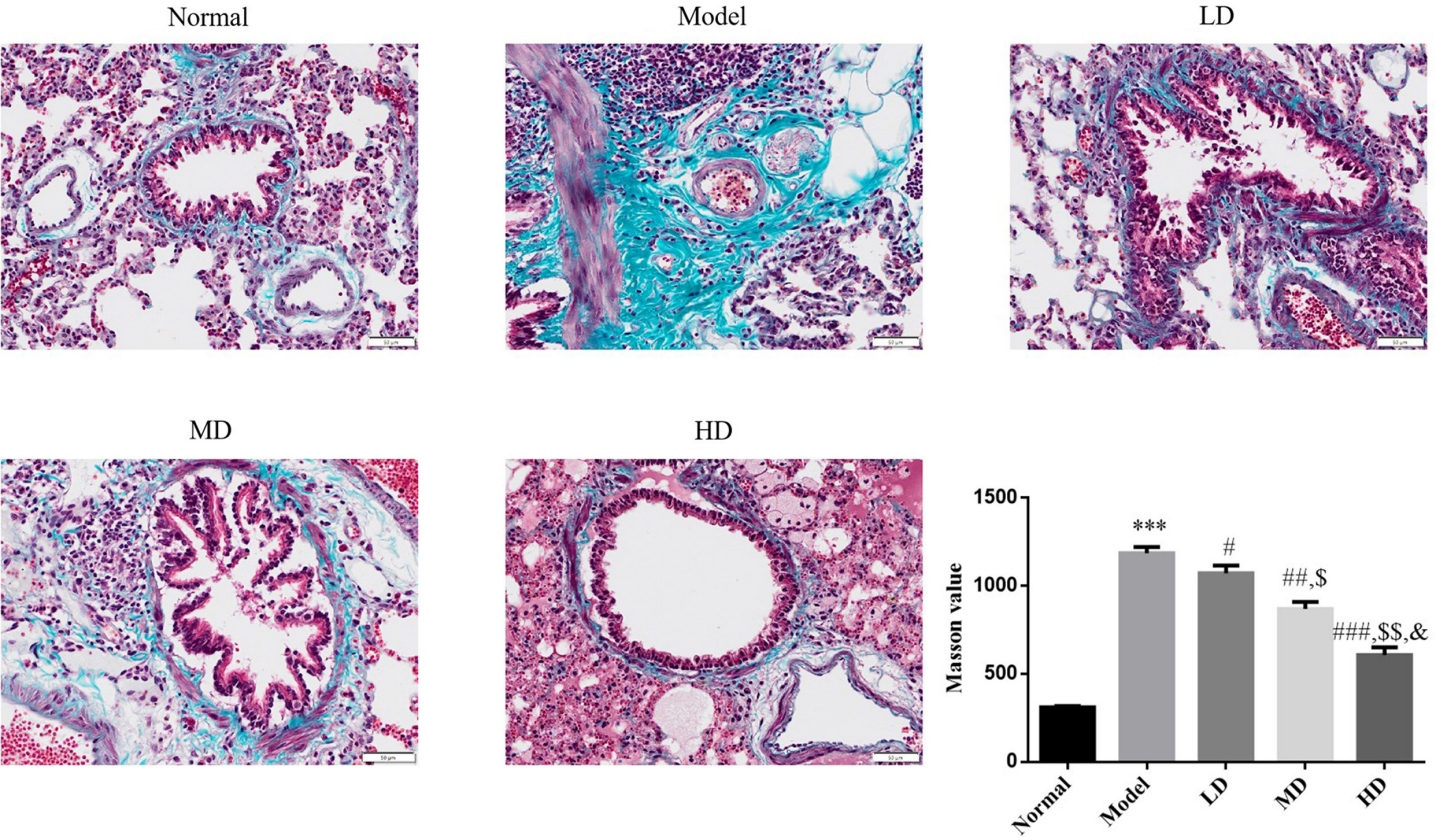
Effect of Vit D on airway collagen deposition (Masson, ×200). Normal: Mice treated with normal saline; Model: asthmatic model mice treated with normal saline; LD: asthmatic model mice treated with low‐dose Vit D (100 IU/kg/day); MD: asthmatic model mice treated with middle‐dose Vit D (500 IU/kg/day); HD: asthmatic model mice treated with high‐dose Vit D (1000 IU/kg/day). ***p < .001, compared with Normal; ^#^ *p* < .05, ^##^ *p* < .01, ^###^ *p* < .001, compared with Model; ^$^ *p* < .05, ^$$^ *p* < .01, compared with LD group; ^&^ *p* < .05, compared with MD group

Compared with the normal control group, the model control group displayed increased muscle and collagen fiber expression in airway mucosal tissues and marked blue collagen deposition. Compared with the model control group, airway subepithelial collagen deposition in Vit D‐treated groups was significantly improved. Quantitative analysis was performed on areas positive for Masson staining, and the airway collagen deposition index was measured. Compared with the normal control group, the airway collagen deposition index was significantly elevated in the model control group (*p* < .001). Vit D treatment significantly reduced the airway collagen deposition index (*p* < .05) in asthmatic mice. The HD Vit D group exhibited a superior effect, as shown in Figure 3.

### 3.5 Effect of Vit D on apoptosis of lung tissue in asthmatic mice model

**FIGURE 4:**
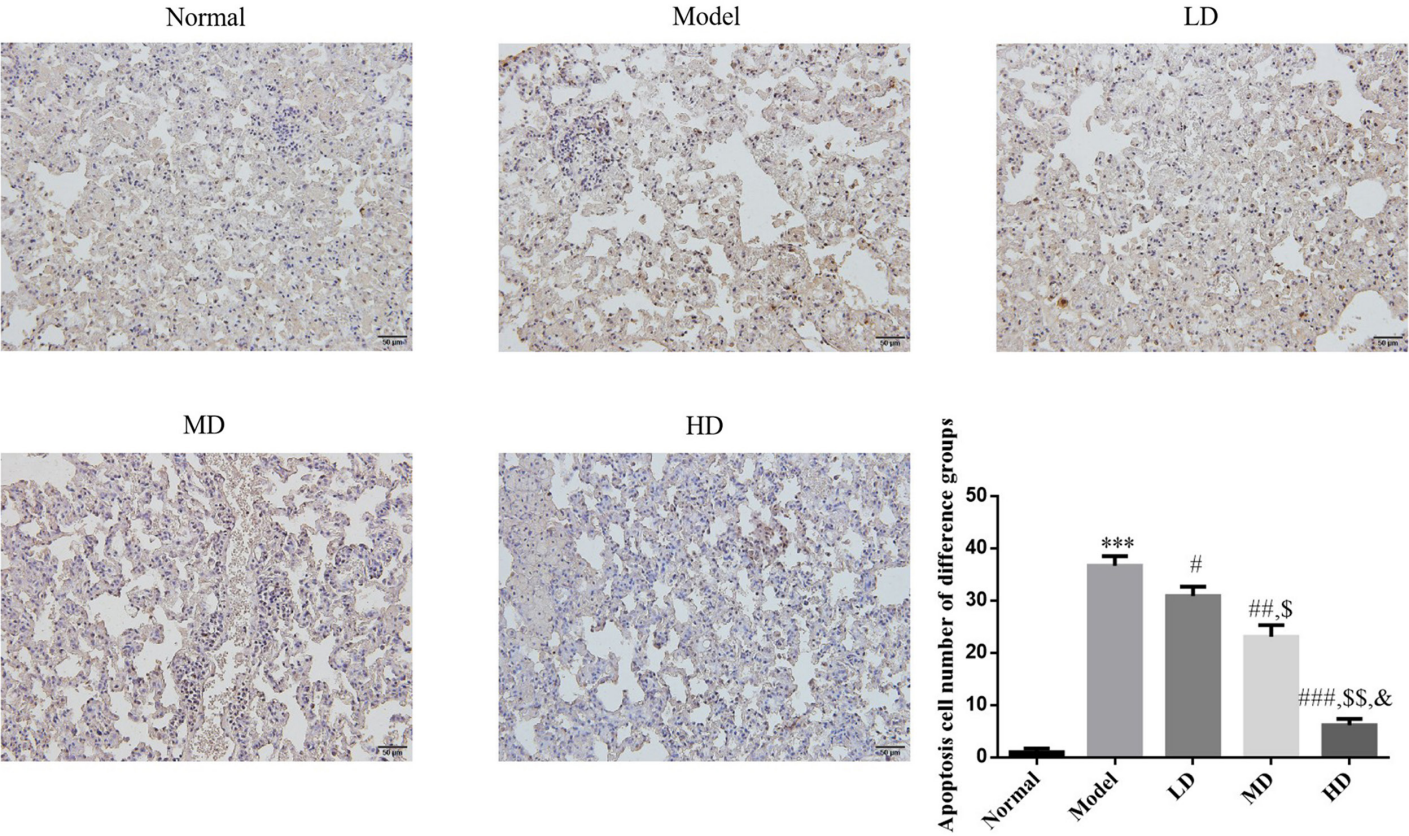
Effect of Vit D on apoptosis cell number in airway mice (TUNEL (terminal deoxynucleotidyl transferase‐mediated deoxyuridine triphosphate (dUTP) nick end‐labeling), ×200). Normal: Mice treated with normal saline; Model: asthmatic model mice treated with normal saline; LD: asthmatic model mice treated with low‐dose Vit D (100 IU/kg/day); MD: asthmatic model mice treated with middle‐dose Vit D (500 IU/kg/day); HD: asthmatic model mice treated with high‐dose Vit D (1000 IU/kg/day). ***p < .001, compared with Normal; ^#^ *p* < .05, ^##^ *p* < .01, ^###^ *p* < .001, compared with Model; ^$^ *p* < .05, ^$$^ *p* < .01, compared with LD group; ^&^ *p* < .05, compared with MD group

Quantitative analysis of apoptotic cells by TUNEL staining revealed that the number of apoptotic cells was significantly increased in the lung tissues of the model group when compared with the normal control group (*p* < .001). Vit D treatment significantly decreased the number of apoptotic cells in Vit D‐treated groups (*p* < .05) when compared with the model group. The HD Vit D exhibited a superior effect, as shown in Figure 4.

### 3.6 Effect of Vit D on expression levels of LC 3B, Beclin1, P62, HIF‐1α, and Notch1 mRNA in asthmatic mice model

**FIGURE 5:**
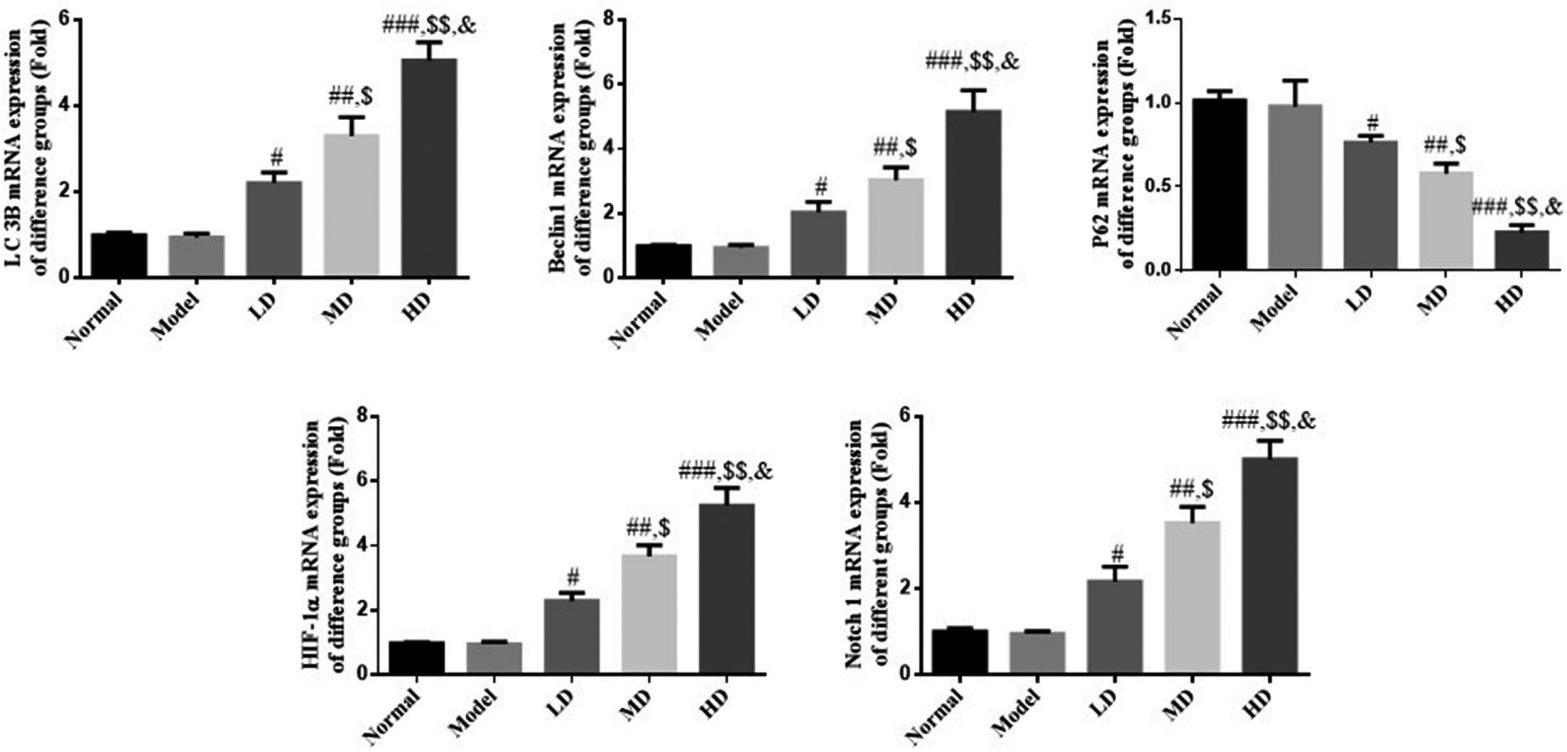
Relative messenger RNA (mRNA) expression determined by reverse‐transcription‐polymerase chain reaction (PCR; RT‐PCR) assay. Normal: Mice treated with normal saline; Model: asthmatic model mice treated with normal saline; LD: asthmatic model mice treated with low‐dose Vit D (100 IU/kg/day); MD: asthmatic model mice treated with middle‐dose Vit D (500 IU/kg/day); HD: asthmatic model mice treated with high‐dose Vit D (1000 IU/kg/day). ***p < .001, compared with Normal; ^#^ *p* < .05, ^##^ *p* < .01, ^###^ *p* < .001, compared with Model; ^$^ *p* < .05, ^$$^ *p* < .01, compared with LD group; ^&^ *p* < .05, compared with MD group

RT‐PCR results revealed that expression levels of LC 3B, Beclin1, P62, HIF‐1α, and Notch1 messenger RNA (mRNA) did not differ significantly between model and normal control groups (*p* > .05). Compared with the model group, mRNA expression levels of LC 3B, Beclin1, HIF‐1α, and Notch1 were significantly increased in Vit D‐treated groups (*p* < .05). The HD Vit D group exhibited a superior effect, as shown in Figure 5.

### 3.7 Effect of Vit D on the expression of LC 3B protein in lung tissue of asthmatic mice model

**FIGURE 6:**
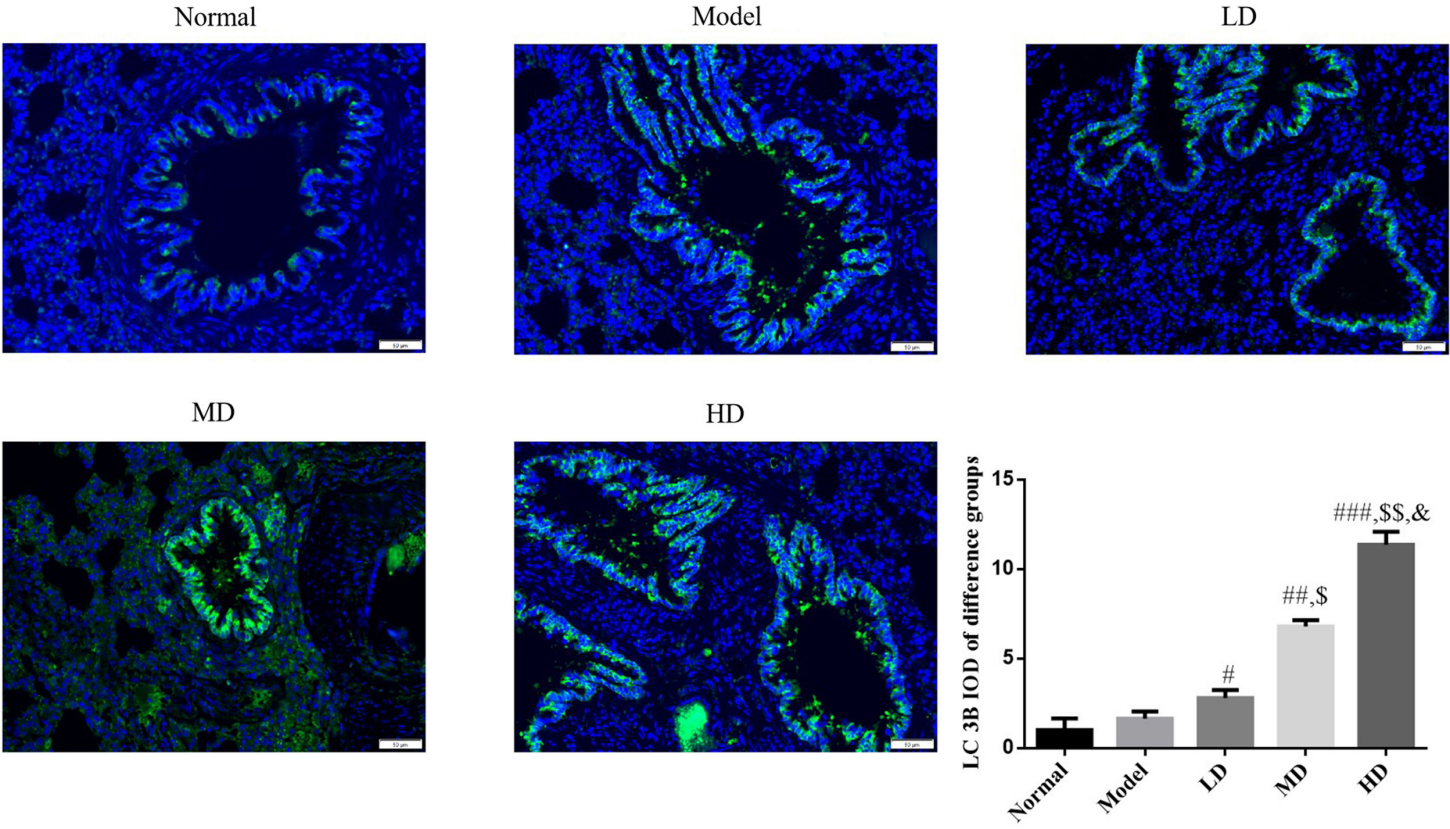
LC 3B (microtubule‐associated protein 1 light chain 3B) expression in different groups (fluorescence immunohistochemistry [FIHC], ×200). Normal: Mice treated with normal saline; Model: asthmatic model mice treated with normal saline; LD: asthmatic model mice treated with low‐dose Vit D (100 IU/kg/day); MD: asthmatic model mice treated with middle‐dose Vit D (500 IU/kg/day); HD: asthmatic model mice treated with high‐dose Vit D (1000 IU/kg/day). ***p < .001, compared with Normal; ^#^ *p* < .05, ^##^ *p* <.01, ^###^ *p* < .001, compared with Model; ^$^ *p* < .05, ^$$^ *p* < .01, compared with LD group; ^&^ *p* < .05, compared with MD group

Based on immunofluorescence findings, LC 3B protein expression did not differ significantly between model and normal control groups (*p* > .05). Compared with the model group, LC 3B protein expression was significantly increased in Vit D‐treated groups (*p* < .05). The HD Vit D group exhibited a superior effect, as shown in Figure 6.

### 3.8 Effect of Vit D on autophagy‐related proteins Beclin1 and P62 in lung tissue of asthmatic mouse model

**FIGURE 7:**
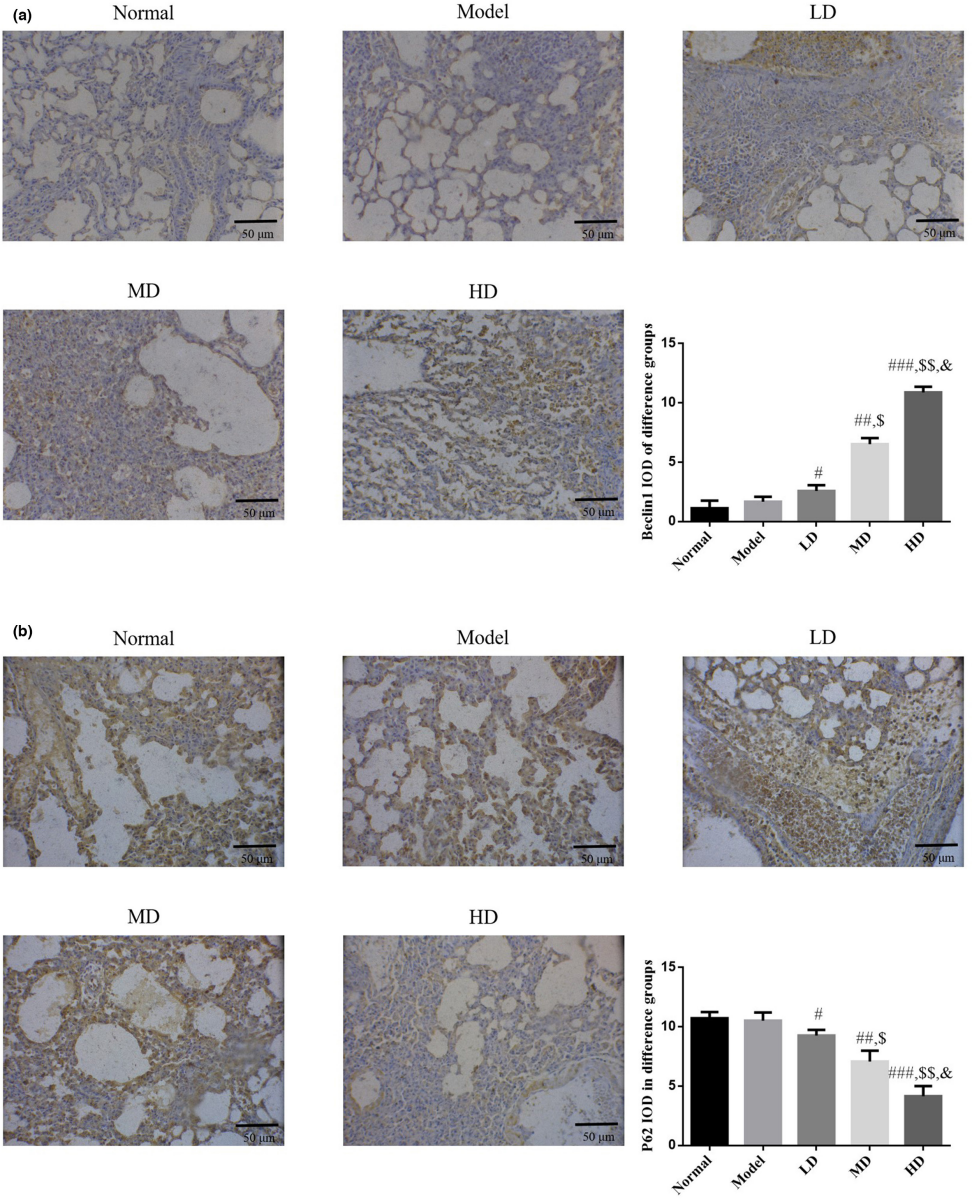
Beclin1 and P62 protein expression in different groups by immunohistochemistry (IHC) (IHC, ×200). Normal: Mice treated with normal saline; Model: asthmatic model mice treated with normal saline; LD: asthmatic model mice treated with low‐dose Vit D (100 IU/kg/day); MD: asthmatic model mice treated with middle‐dose Vit D (500 IU/kg/day); HD: asthmatic model mice treated with high‐dose Vit D (1000 IU/kg/day). (a) Beclin1 protein expression in different groups. (b) P62 protein expression in different groups. ***p < .001, compared with Normal; ^#^ *p* < .05, ^##^ *p* < .01, ^###^ *p* < .001, compared with Model; ^$^ *p* < .05, ^$$^ *p* < .01, compared with LD group; ^&^ *p* < .05, compared with MD group

According to immunohistochemical test results, protein expression levels of Beclin1 and P62 did not differ significantly between model and normal control groups (*p* > .05). Compared with the model group, the expression of LC 3B protein was significantly increased in Vit D‐treated groups (*p* < .05), while the expression of P62 protein was significantly decreased (*p* < .05). The HD Vit D group exhibited a superior effect, as shown in Figure 7.

### 3.9 Effect of Vit D on HIF‐1α and Notch1 protein expression in lung tissue of asthmatic mice model

**FIGURE 8:**
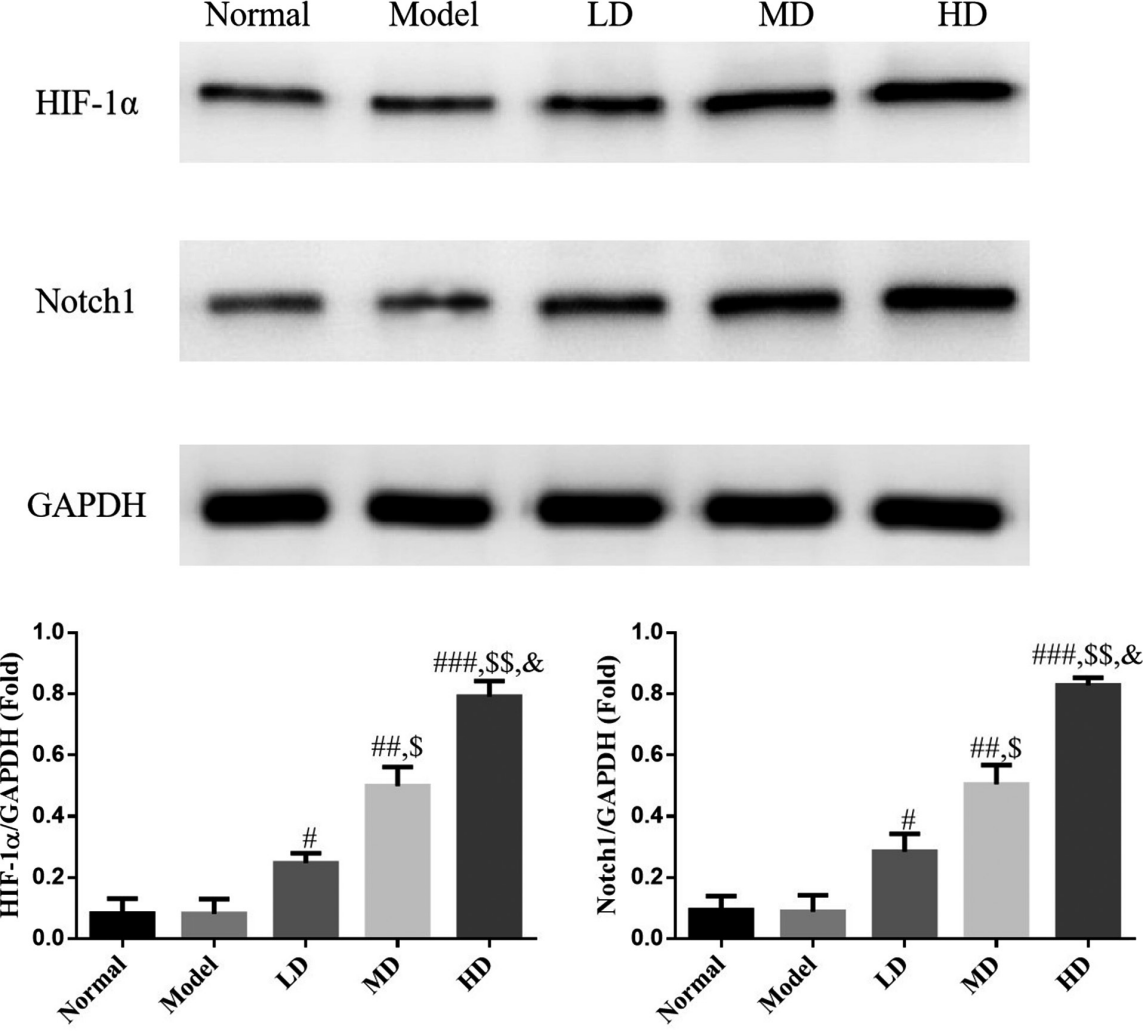
HIF‐1α (hypoxia‐inducible factor 1α) and Notch1 (neurogenic locus notch homolog protein 1) expression by western blotting analysis. Normal: Mice treated with normal saline; Model: asthmatic model mice treated with normal saline; LD: asthmatic model mice treated with low‐dose Vit D (100 IU/kg/day); MD: asthmatic model mice treated with middle‐dose Vit D (500 IU/kg/day); HD: asthmatic model mice treated with high‐dose Vit D (1000 IU/kg/day). ***p < .001, compared with Normal; ^#^ *p* < .05, ^##^ *p* < .01, ^###^ *p* < .001, compared with Model; ^$^ *p* < .05, ^$$^ *p* < .01, compared with LD group; ^&^ *p* < .05, compared with MD group

Based on western blotting analysis, HIF‐1α and Notch1 protein expression levels did not differ significantly between the model and control groups (*p* > .05). Compared with the model group, protein expression levels of HIF‐1α and Notch1 were significantly increased in Vit D‐treated groups (*p* < .05), with the HD Vit D group exhibiting a superior effect, as shown in Figure 8.

## 4 DISCUSSION

Bronchial asthma is an airway chronic inflammatory disease involving various cells, including inflammatory and structural cells, as well as cellular components (Hyland et al., 2018). Chronic inflammation can induce airway hyperresponsiveness and reversible airflow restriction, resulting in repeated wheezing, shortness of breath, chest tightness, cough, and other symptoms (Yan, 2018). As the current first‐line therapy, glucocorticoids are the cornerstone of modern asthma treatment. Despite their effective regulation of chronic inflammation and immune disorders in asthma (Girodet et al., 2011), studies have shown that only long‐term and high‐dose corticosteroid administration can improve existing airway remodeling changes. However, hormone therapy can cause substantial systemic adverse reactions, limiting its clinical application for treating asthma (Feltis et al., 2007; Wang et al., 2008). The other commonly used drugs, such as leukotriene antagonists, long‐ and short‐acting β‐receptor agonists, and immunoglobulin E (IgE) monoclonal antibodies, have failed to achieve satisfactory results during asthma (Girodet et al., 2011). Therefore, it is important to explore and identify more effective drugs for treating asthma.

In the present study, our findings revealed that mice in the model group exhibited increased airway responsiveness and inflammatory cell infiltration into the blood vessels of lung tissue and surrounding bronchus, demonstrating the successful establishment of the asthma model. Meanwhile, Vit D can reduce airway hyperresponsiveness and lung tissue damage in asthmatic mice. Th17 cells can reportedly promote T cell activation and stimulate immune cells to produce diverse cytokines, among which IL‐17 and IL‐6 markedly impact the occurrence of asthma (Ramakrishnan et al., 2019). IL‐10 inhibits airway inflammation by directly inhibiting inflammatory cell activation and proliferation, cytokine secretion, T cell response, and specific IgE secretion (Branchett et al., 2020). Herein, we observed that Vit D significantly decreased expression levels of IL‐6 and IL‐17, whereas levels of the asthma‐protective factor IL‐10 were increased. Repeated airway inflammation can cause structural changes in the airway wall, including pathological changes such as airway epithelial cell injury and shedding, goblet cell metaplasia, and wall thickening, ultimately resulting in airway remodeling (Boulet, 2018; Chen et al., 2018; Cheng et al., 2018). Our findings revealed that Vit D could alleviate airway inflammation and remodeling, as well as reduce airway hyperresponsiveness in RSV‐induced asthmatic mice.

Autophagy regulates the replication and proliferation of viruses in host cells, supports the interaction between antigen‐presenting cells and T cells during viral infection, promotes DC activation and maturation, and is an important pathway for protecting the human body against virus invasion. This contradictory biological effect may be associated with the specificity of the infecting virus and the immune microenvironment of the human body (Seto et al., 2013). Studies have confirmed that several genes are involved and regulate the pathogenesis of asthma, including some autophagy‐related genes, such as autophagy‐related 5 (ATG5) and sequestosome 1 (SQSTM1; Kasembeli et al., 2018). In the present study, immunofluorescence and immunohistochemistry results revealed that autophagy marker proteins LC 3B (Abdel Karim et al., 2019), Beclin1 (Chen et al., 2020), and P62 (Li et al., 2015) were unaltered in lung tissues of RSV‐infected mice; however, expression levels of LC 3B and Beclin1, both at the gene and protein levels, were significantly increased following Vit D treatment. The P62 gene and protein levels were significantly decreased, suggesting that the mechanism of Vit D in treating RSV‐induced asthma may involve enhanced autophagy in lung tissue.

HIF‐1α, a transcription factor in cells, forms a heterodimer with hypoxia‐inducible factor 1β (HIF‐1β), which can regulate the expression of more than 100 target genes, and plays an important role in the process of regulating blood vessel formation, placental and embryo development, glucose metabolism, apoptosis, and autophagy (Lee et al., 2004). It has been confirmed that HIF‐1α is positively regulated by Notch1 (Branco et al., 2019; Yu et al., 2019). In the present study, Vit D treatment enhanced Notch1–HIF‐1α expression in lung tissue, as well as accelerated autophagy, reduced airway hyperreactivity, and alleviated airway inflammation and remodeling; these findings indicated that facilitating Notch1–HIF‐1α expression might be an important target for Vit D‐mediated regulation of autophagy during treatment of RSV‐induced asthma.

Herein, an RSV‐induced asthma mouse model was successfully established. Our findings demonstrated that Vit D could reduce airway responsiveness, alleviate lung tissue damage, and improve airway inflammation, which may be related to the promotion of Notch1–HIF‐1α protein expression and positive regulation of autophagy. Nevertheless, the drawbacks of the present study need to be addressed. An in‐depth assessment of the Vit D‐mediated therapeutic mechanism in asthmatic mice is lacking. Accordingly, further studies elucidating the underlying mechanism of Vit D in asthmatic mice will be performed at a molecular level.

## Data Availability

Research data are not shared.
